# Corrigendum: Subsurface Microbial Habitats in an Extreme Desert Mars-Analog Environment

**DOI:** 10.3389/fmicb.2019.02129

**Published:** 2019-09-18

**Authors:** Kimberley A. Warren-Rhodes, Kevin C. Lee, Stephen D. J. Archer, Nathalie Cabrol, Linda Ng-Boyle, David Wettergreen, Kris Zacny, Stephen B. Pointing, NASA Life in the Atacama Project Team

**Affiliations:** ^1^NASA Ames Research Center, Mountain View, CA, United States; ^2^The SETI Institute, Mountain View, CA, United States; ^3^School of Sciences, Auckland University of Technology, Auckland, New Zealand; ^4^College of Engineering, University of Washington, Seattle, WA, United States; ^5^Institute of Robotics, Carnegie Mellon University, Pittsburgh, PA, United States; ^6^Honeybee Robotics Spacecraft Mechanisms Corp., Pasadena, CA, United States; ^7^Yale-NUS College, National University of Singapore, Singapore, Singapore; ^8^Department of Biological Sciences, National University of Singapore, Singapore, Singapore

**Keywords:** Atacama, desert soil, mars, moisture stress, soil bacteria

One of the author's name was incorrect in the Funding Statement. “Cecilia Demargasso” should be “Cecilia Demergasso.”

The authors apologize for this error and state that this does not change the scientific conclusions of the article in any way. The original article has been updated.

