# Supplementary material for: Subsurface Microbial Habitats in an Extreme Desert Mars-Analog Environment
Source: Front Microbiol. 2019 Feb 28;10:69. doi: 10.3389/fmicb.2019.00069 (PMC6403490; doi:10.3389/fmicb.2019.00069)
Supplement: Supplementary file 1 [file Data_Sheet_1.pdf]

Subsurface microbial habitats in an extreme desert Mars-analogue environment

Online Supplementary Material

Figure S1. a) Sampling transect in the hyper-arid core of the Atacama Desert, circles along the transect line indicate sampling locations, transect line = 50km (image: Google Earth); b) Schematic indicating relative location of the eleven sampling sites (not to scale). The drill was not deployed for microbiology sampling at sites 3,4,5,7 of the traverse due to terrain and operational constraints on sample recovery. GPS coordinates for each sampling site were: Site 1, 24.76853 S, 69.65059 W, alt. 2053m; Site 2, 24.7721 S, 69.64957 W, alt. 2066m; Site 6, 24.7878 S, 69.60629 W, alt. 2314m; Site 8, 24.63488 S, 69.45376 W, alt. 1984m; Site 9, 24.63493 S, 69.45411 W, alt. 1990m; Site 10, 24.6343 S, 69.45417 W, alt. 1991m; Site 11, 24.63435 S, 69.45195 W, alt. 1988m.

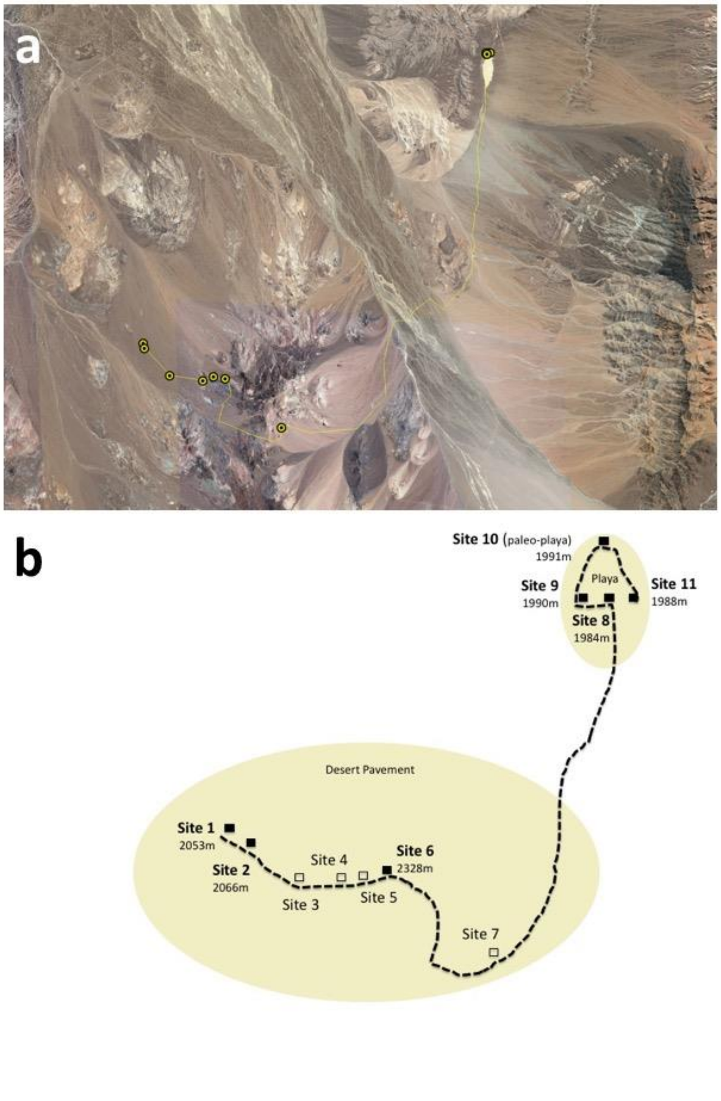

Figure S2. The Carnegie Mellon Zöe rover deployed in the Atacama Desert: a) rover showing solar panel dorsal surface (i), drill apparatus (ii), navigation (iii), rover body housing sample analysis and control systems (iv}, arrow indicates direction of travel; b) rover deployed next to a human subject for scale (images: Carnegie Mellon University/NASA).

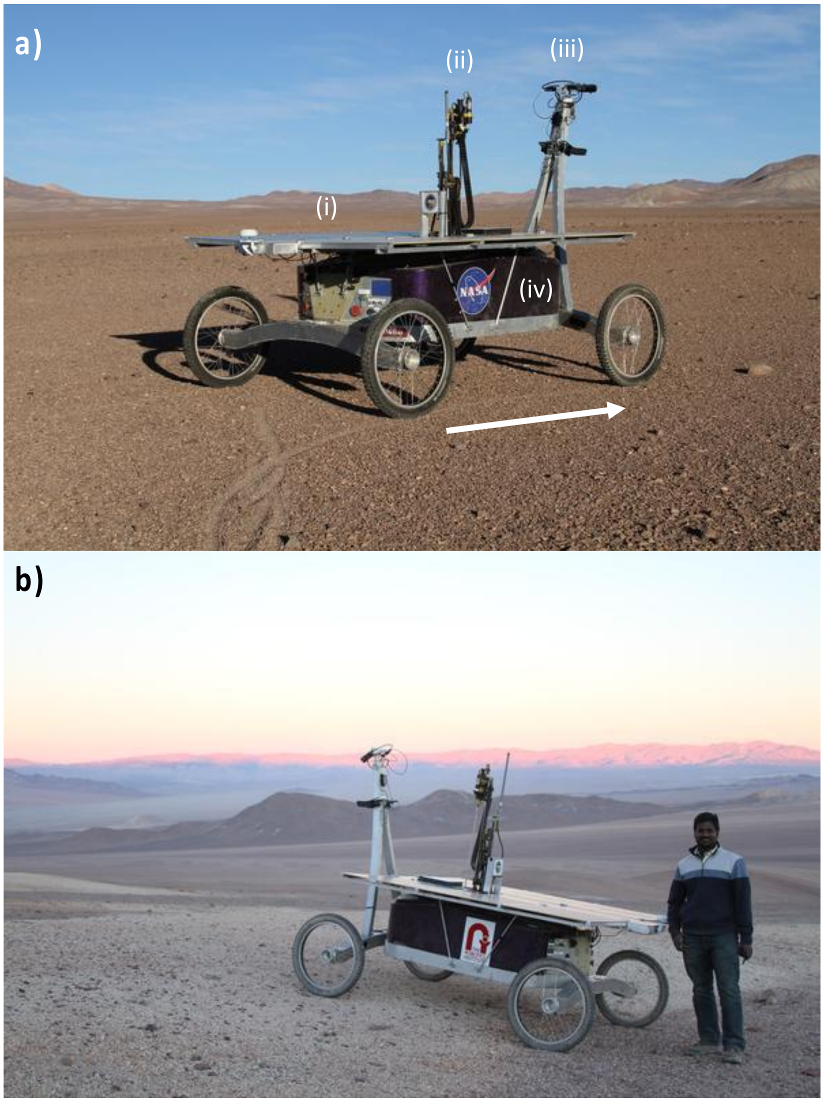

Figure S3. The Zöe rover sample recovery and analysis payload: a) drill assembly in the disassembled rover body; b) drill and sample carousel assembly in the disassembled rover body (images: Carnegie Mellon University/NASA), c) drill being deployed in Atacama sediment (image: Kris Zacny).

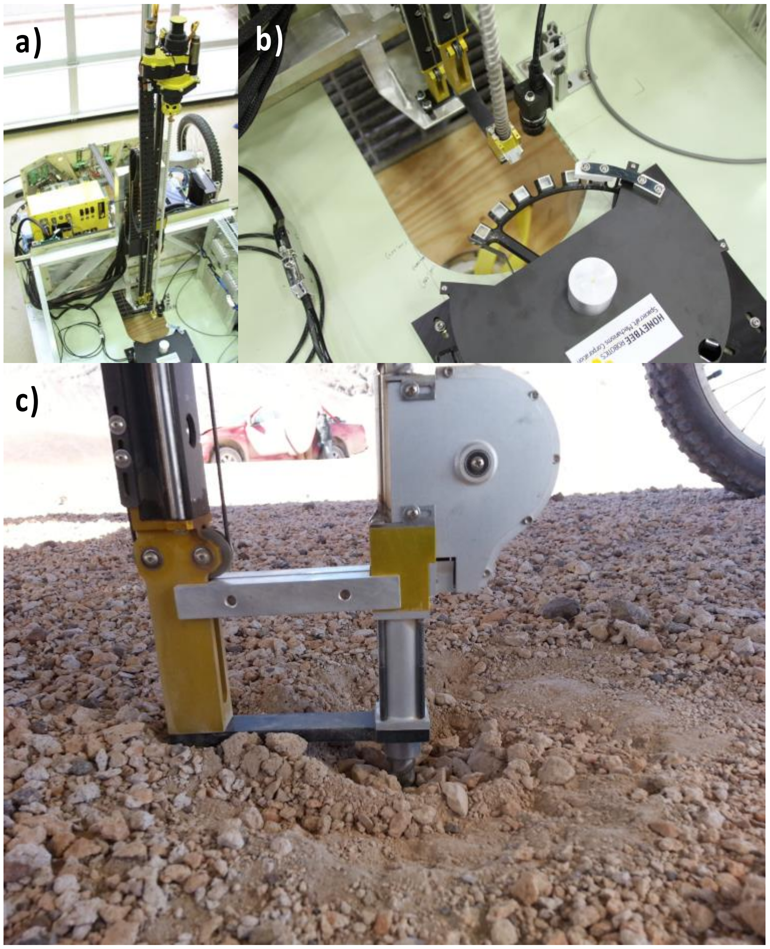

Figure S4. Major trends in geochemical variables within subsurface sediments, red symbols = desert pavement; blue symbols = playa. Data for manually collected samples shown here. Variables that did not vary significantly with depth are not plotted. Trend lines were plotted using the Loess Method. Shaded area represents 95% confidence intervals.

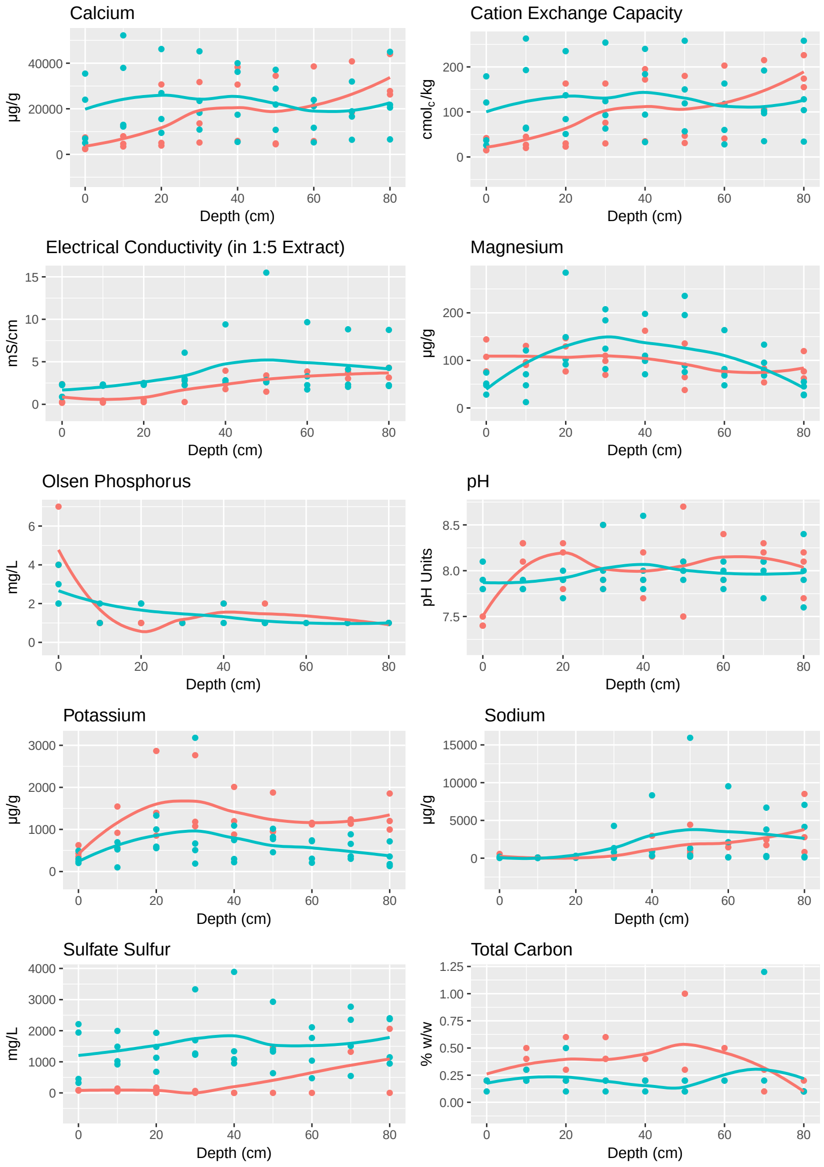

Figure S5. Canonical Correspondence Analysis (CCA) triplot with symmetrical scaling indicating differences in sediment geochemistry with terrain and depth as measured in real-time by the MMRS payload on-board the autonomous rover and validated by laboratory Raman spectroscopy.

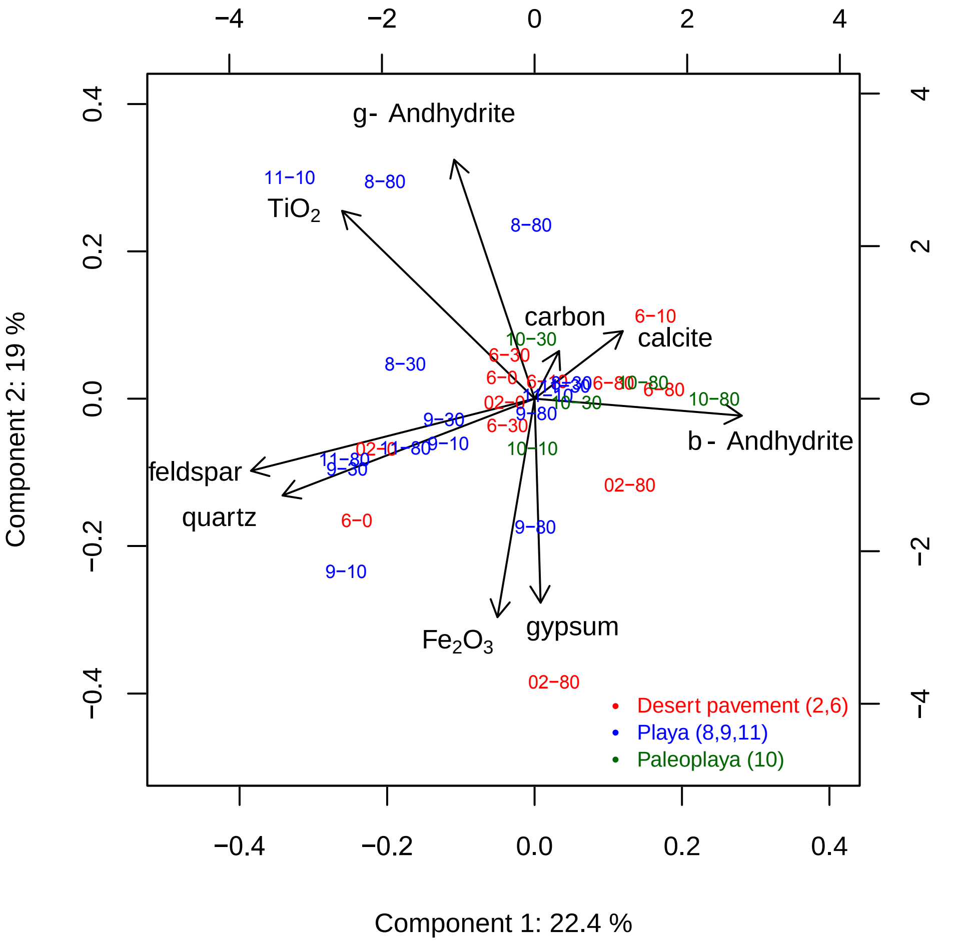

Figure S6. Summary plot of temporal trend in sediment temperature and moisture (as relative humidity, RH) in subsurface depth horizon at representative desert pavement site 1. The dataloggers were installed at various depths in manually excavated pit during sample collection for this study, the pits were then backfilled and dataloggers recovered after 14 months recording data *in situ*. A) 10 cm depth, b) 30 cm depth, c) 80 cm depth. The "spike" in RH in June 2014 was due to a rainfall event.

a) 10cm depth

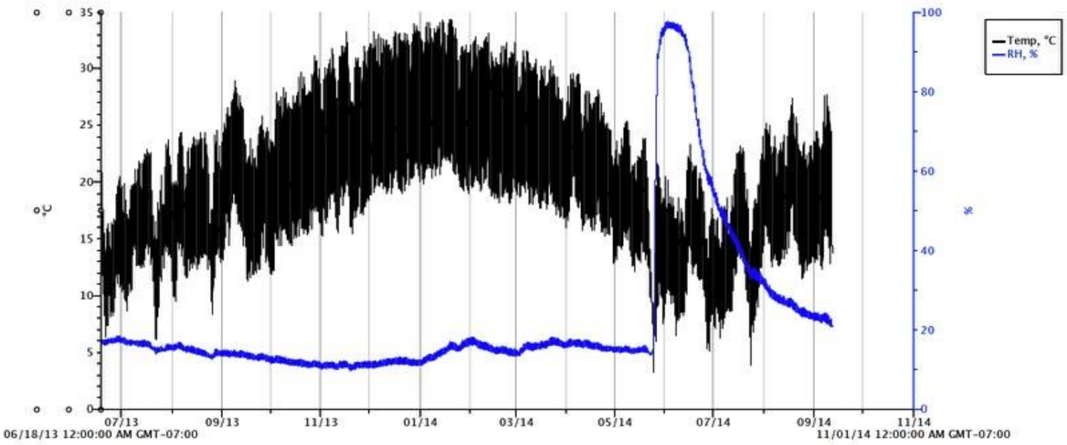

b) 30 cm depth

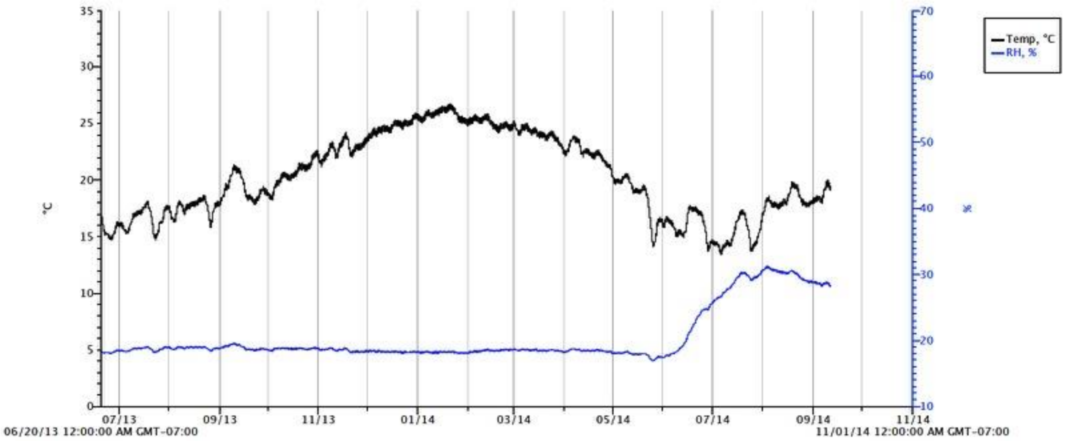

c) 80 cm depth

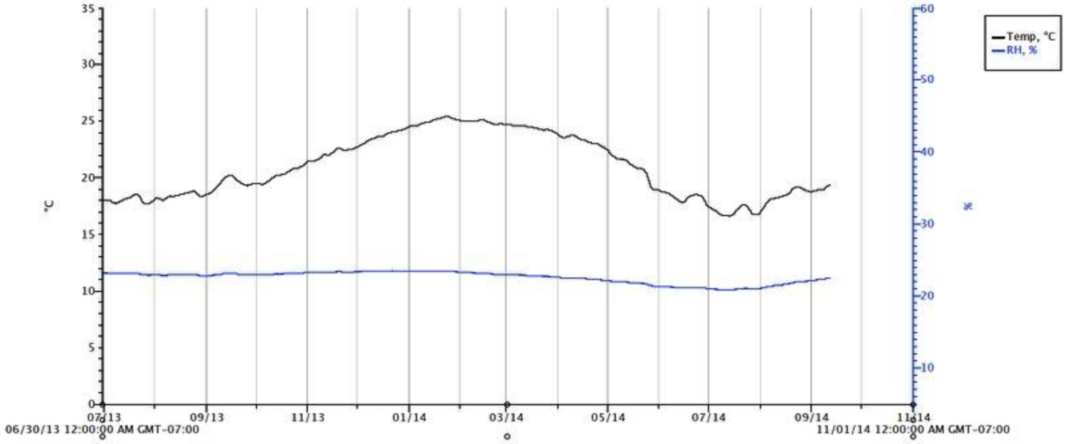
Temp, °C
RH, %
°C
%
35
30
25
20
15
10
5
0
60
50
40
30
20
10
07/13
09/13
11/13
01/14
03/14
05/14
07/14
09/14
11/14
06/30/13 12:00:00 AM GMT-07:00
11/01/14 12:00:00 AM GMT-07:00

Figure S7. Major trends in geochemical variables correlating with bacterial biosignatures in subsurface sediments, red symbols = desert pavement; blue symbols = playa. Data for manually collected samples shown here. Variables that did not vary significantly with depth are not plotted. Trend lines were plotted using the Loess Method. Shaded area represents 95% confidence intervals.

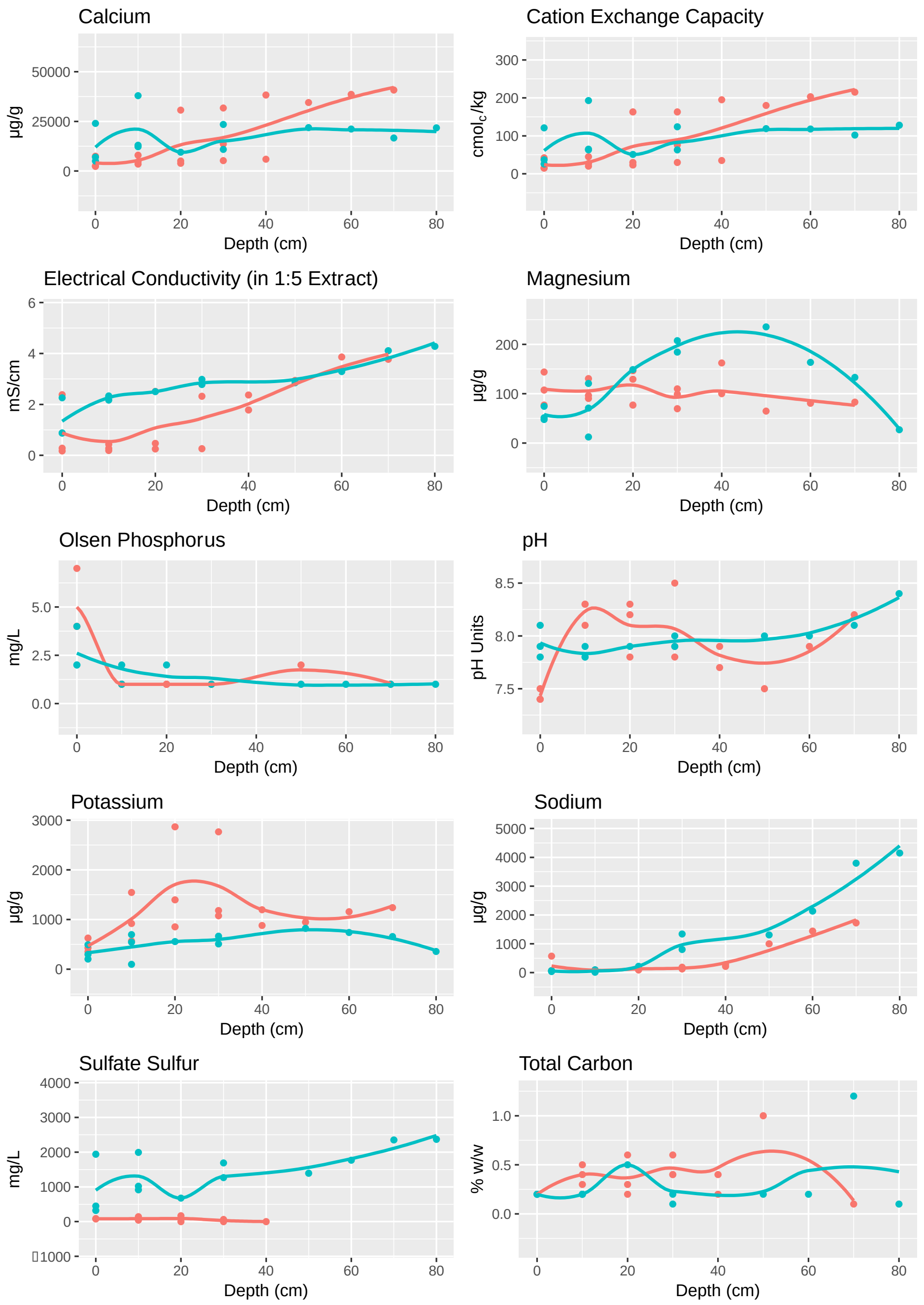

Figure S8. Shannon's Diversity estimates (H) for bacterial diversity in vertical sediment horizons from manual recovery (a) and rover recovery (b). Orange symbols = desert pavement; green symbols = playa. Grey shaded area indicates limit for 95% Confidence Intervals.

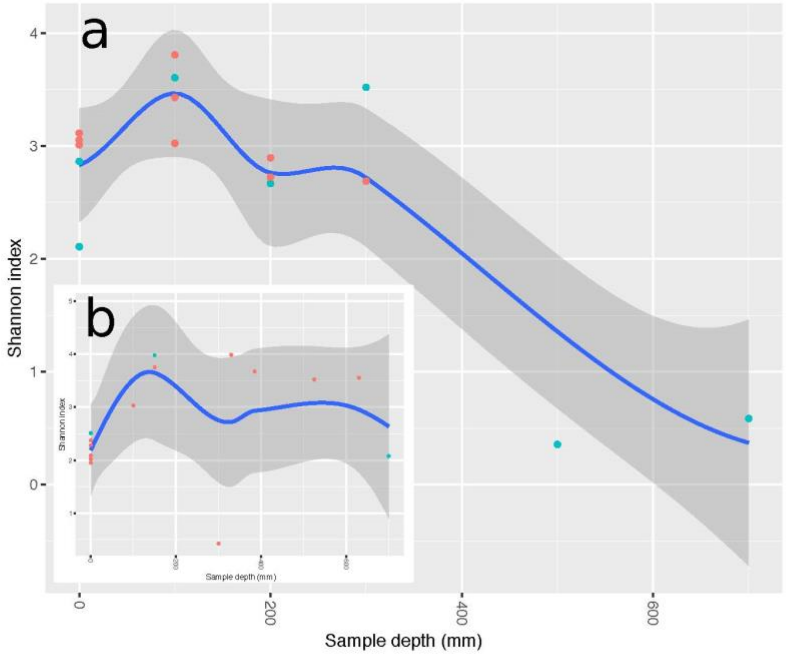

Figure S9. Distribution of bacterial diversity by taxonomic class with sediment depth for manual (M) and autonomous rover drill (D) recovered samples yielding amplifiable DNA (see Fig. S5). The numeric suffix after each M and D sample code denote the depth or depth range for samples recovered. Coloured shading indicates relative abundance within each community for a given bacterial class. Sequencing read depth for each sample is given in Table S6.

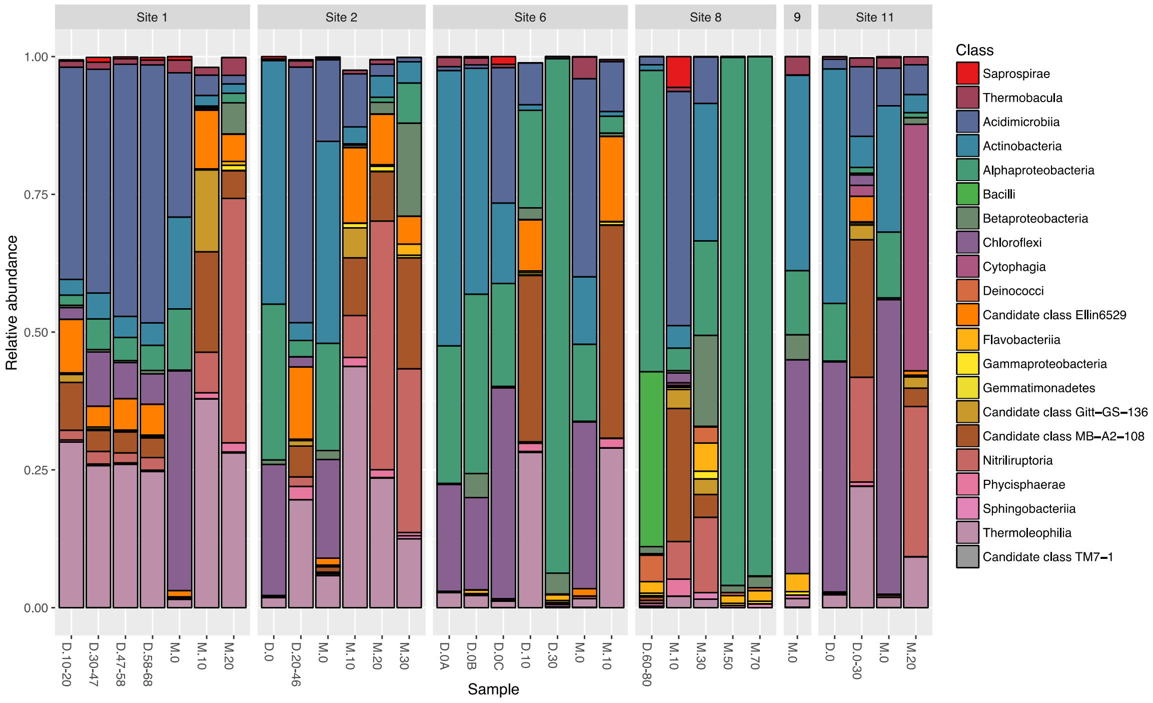

Table S1. Comparison of previous microbial diversity estimates for <u>surface-associated</u> hyper-arid sediment in the Atacama Desert (data for arid and semi-arid locations are not included if part of these studies).

Ref. in main text	Year	Samples	Depth (cm)	Cultivation	Biochemical	Environmental DNA
(1)	2003	1	0-10	10^3-10^6 cfu/g sediment; rRNA phylotypes of cultivated strains: Low G-C Gram positives (Bacillus) Actinobacteria (*Blastococcus/Geodermatophilus; Streptomyces)*	Negative for biological activity assayed by labelled substrate release	N/A
(2)	2006	33	Surface [plus 3 soil pits sampled to 80 cm (n=2) or 90 cm (n=1)]	<10^5 cfu/g rRNA gene sequences indicated *Blastococcus* sp. Dominated cultivable taxa. Only 1 of 18 sub-surface samples yielded cultivable bacteria <10^2 cfu/g.	N/A	N/A
(3)	2006	1	25-30	Unspecified cultivable bacteria	N/A	DGGE fingerprinting: Actinobacteria, Gammaproteobacteria, Gemmatimonadetes, Planctomycetes
(4)	2007	7	2-20	10^2-10^3 cfu/g sediment	PLFA estimate: 10^5-10^6 cells/g Corresponding to Actinobacteria, Firmicutes, Proteobacteria	Clone libraries: 6 taxa from four phyla: Actinobacteria, Firmicutes, Proteobacteria, Thermi
(5)	2007	3	0-15	10^3 cfu/g sediment; rRNA phylotypes of cultivated strains: *Bacillus*; Bradyrhizobiaceae; *Rhodopseudomonas*	PLFA estimate: 10^6-10^7 cells/g	N/A
(6)	2013	45	0-10	10^3 cfu/g sediment	Positive for labelled substrate mineralization in sediment microcosms	Pyrosequencing (library sizes 200-1000 filtered reads), 14 phyla: Actinobacteria 72-88%, Acidobacteria 3.8-6.6%, Proteobacteria 2.2-9.2%
(7)	2018	6	0-30 [the study also included 3 sub-surface samples, at 50 cm (n=1) and 100 cm (n=2} taken after a rain event]	<10^2 16S rRNA gene copies/g sediment estimated using qPCR	10^4-10^5 endospores/g sediment (as dipicolinic acid); positive ATP, flourescein diacetate hydrolysis, PLFA assays	Illumina sequencing (unspecified filtered library sizes) 19 bacterial phyla: highly variable, near surface dominated by Actinobacteria, at depth dominated by Firmicutes and Proteobacteria. Also reports 11 fungal phyla spanning ascomycota and basicidomycota

References

1. Navarro-Gonzalez R, et al. (2003) Mars-like soils in the Atacama Desert, Chile, and the dry limit of microbial life. *Science (80-)* 302(5647):1018–1021.
2. Bagaley DR (2006) Uncovering bacterial diversity on and below the surface of a hyper-arid environment, The Atacama Desert, Chile. Dissertation (Louisiana State University).
3. Drees KP, et al. (2006) Bacterial community structure in the hyperarid core of the Atacama Desert, Chile. *Appl Env Microbiol* 72(12):7902–7908.
4. Connon SSA, Lester EDE, Shafaat HHS, Obenhuber DC, Ponce A (2007) Bacterial diversity in hyperarid Atacama Desert soils. *J Geophys Res - Biogeosci* 112(G4):G04S17.
5. Lester ED, Satomi M, Ponce A (2007) Microflora of extreme arid Atacama Desert soils. *Soil Biol Biochem* 39(2):704–708.
6. Crits-Christoph A, et al. (2013) Colonization patterns of soil microbial communities in the Atacama Desert. *Microbiome* 1(1):28.
7. Schulze-Makuch D, et al. (2018) Transitory microbial habitat in the hyperarid Atacama Desert. *Proc Natl Acad Sci U S A* 115(11):2670–2675.

Table S2. Mineral distribution estimated by laser Raman spectroscopy as measured in real-time by the on-board the autonomous rover (MMRS) and laboratory validation (Lab). All values are relative abundance estimates.

Site	Depth (cm)	Raman method	gypsum	*b*-andhydrite	g-anhydrite	calcite	quartz	feldspar	carbon
2	0	MMRS	0	0	0	0	3	7	0
	0	Lab	0	0	0	0	14	13	0
	80	MMRS	21.5	13	0	0	0	0	0
	80	Lab	21.5	12	0	2	2	4	0
6	0	MMRS	0	0	0	0	2	6	0
	0	Lab	0	0	0	0	9	18	0
	10	MMRS	0	0	0	32	1	0	0
	10	Lab	0	0	0	7	3	4	0
	30	MMRS	4	0	0	1	8.5	2.5	0
	30	Lab	0	0	0.5	2.5	5	4	0
	80	MMRS	0	28	0	0	1.5	0	0
	80	Lab	0	11.5	0	0	0	1	0
8	30	MMRS	0	0	0	0	2	0	0
	30	Lab	2	0	0	4	7	10	0
	80	MMRS	1	3.5	1.5	1	3	0	1
	80	Lab	2	2	2	1.5	5	7	0
9	10	MMRS	2	0	0	0	19	0.5	0
	10	Lab	8	0	0	0	18	12	0
	30	MMRS	9	0	0	0	9	5	0
	30	Lab	8	0	0	0	12	16	0
	80	MMRS	27	0	0	0	4	3	0
	80	Lab	10	0	0	0	10	0	2
10	0	MMRS	0	0	0	0	1	0	0
	0	Lab	6	3	1	0	1	1	0
	10	MMRS	7	0	0	nd	1	1	0
	10	Lab	11	0	0	1	4.5	3	0
	30	MMRS	4.5	1	0	1	1	1	0
	30	Lab	7	3	1	3	3	3	0
	80	MMRS	0	18	0	0	0	0	0
	80	Lab	0	47	0	0	3	0	0
11	10	MMRS	1	0	0	0	3	2	0
	10	Lab	0	0	1	0	6.5	9	0
	30	MMRS	0	0	0	0	3	0	0
	30	Lab	1	0	1	0	11	2	0
	80	MMRS	11	0	0	0	17	3	0
	80	Lab	5	0	0	0	16	13	0

Table S3. Sediment geochemistry and DNA yield for desert pavement and playa depth horizons (italicized entries indicate DNA also recovered using autonomous rover sampling, shown in Fig. S4). Dashed lines indicate value not detected. Y = yes, N = No, ND = not detected, NT or - = not tested.

Site	Site type	Sediment depth (cm)	DNA yield (ng/g)	Sequence data aquired (Y/N), Italic = DNA also from rover samples	Bulk density (g/mL)	pH (pH units)	Electrical conductivity (EC) (mS/cm)	Soluble salts (%)	C/N Ratio	Total carbon (% w/w)	Total nitrogen (% w/w)	Olsen phosphorus (mg/L)	Sulfate sulfur (mg/kg)	Extractable organic sulphur (mg/kg)	Anion storage capacity (%)	Cation exchange capacity (meq/100g)	Potassium (meq/100g)	Potassium (µg/g)	Calcium (meq/100g)	Calcium (µg/g)	Magnesium (meq/100g)	Magnesium (µg/g)	Sodium (meq/100g)	Sodium (µg/g)
1	Desert Pavement	0	3.56	Y	1.27	7.5	0.28	0.1	9.2	<0.1	<0.04	4	88	<2	<15	15	0.9	351.9	12.8	2560	0.88	107.36	0.29	66.7
1	Desert Pavement	10	3.08	Y	1.26	8.3	0.19	0.07	22.3	0.3	<0.04	1	43	<2	<15	27	2.35	918.85	23.2	4640	0.79	96.38	0.39	89.7
1	Desert Pavement	20	<0.067	Y	1.29	8.3	0.25	0.09	9	0.3	<0.04	1	99	<2	<15	30	3.57	1395.87	25.2	5040	1.06	129.32	0.66	151.8
1	Desert Pavement	30	<0.067	*Y*	1.26	7.9	2.32	0.81	40.9	0.4	<0.04	<1	-	-	-	163	2.75	1075.25	158.6	31720	0.9	109.8	0.65	149.5
1	Desert Pavement	40	<0.067	*Y*	1.41	7.7	2.37	0.83	33.7	0.2	<0.04	<1	-	-	-	195	2.25	879.75	191.4	38280	0.82	100.04	0.94	216.2
1	Desert Pavement	50	<0.067	*Y*	1.34	7.5	2.94	1.03	16.2	1	0.06	2	-	-	-	180	2.42	946.22	172.4	34480	0.53	64.66	4.34	998.2
1	Desert Pavement	60	<0.067	*Y*	1.46	7.9	3.86	1.35	3.4	<0.1	<0.04	<1	-	-	-	203	2.96	1157.36	193	38600	0.66	80.52	6.26	1439.8
1	Desert Pavement	70	<0.067	*Y*	1.44	8.2	3.77	1.32	3	0.1	<0.04	1	-	-	-	215	3.17	1239.47	204	40800	0.68	82.96	7.5	1725
1	Desert Pavement	80	<0.067	N	1.48	8.1	4.31	1.51	3	0.2	0.06	1	-	-	-	155	3.07	1200.37	139	27800	0.51	62.22	12.08	2778.4
2	Desert Pavement	0	<0.067	Y	1.49	7.4	2.38	0.83	21.3	0.2	<0.04	4	-	-	-	42	1.6	625.6	37.3	7460	0.63	76.86	2.48	570.4
2	Desert Pavement	10	3.28	Y	1.12	8.3	0.27	0.09	12.6	0.4	<0.04	1	69	<2	<15	20	1.45	566.95	17.3	3460	0.74	90.28	0.28	64.4
2	Desert Pavement	20	<0.067	Y	1.37	8.2	0.47	0.16	10.8	0.2	<0.04	1	170	<2	<15	23	2.18	852.38	19.3	3860	0.63	76.86	0.39	89.7
2	Desert Pavement	30	<0.067	Y	1.34	8.5	0.26	0.09	67.4	0.6	<0.04	1	62	<2	24	30	3.02	1180.82	26	5200	0.81	98.82	0.55	126.5
2	Desert Pavement	40	<0.067	*Y*	1.55	7.9	1.78	0.62	40.4	0.4	<0.04	<1	0	-	-	35	3.06	1196.46	29.6	5920	1.33	162.26	1.21	278.3
2	Desert Pavement	50	<0.067	N	1.48	8.1	1.48	0.52	37.9	0.3	<0.04	1	0	-	-	31	2.45	957.95	24	4800	1.11	135.42	2.97	683.1
2	Desert Pavement	60	<0.067	N	1.53	8.4	-	-	28.3	0.5	<0.04	1	0	-	-	41	2.86	1118.26	29.3	5860	0.6	73.2	8.5	1955
2	Desert Pavement	70	<0.067	N	1.49	8.3	3.03	1.06	18.8	0.3	<0.04	<1	1,321	<2	<15	106	2.9	1133.9	92.3	18460	0.44	53.68	10.62	2442.6
2	Desert Pavement	80	<0.067	N	1.32	7.7	3.14	1.1	3.2	0.1	0.04	<1	2,060	<2	<15	226	2.55	997.05	220	44000	0.63	76.86	3.57	821.1
6	Desert Pavement	0	6.507	Y	1.42	7.4	0.17	0.06	6.7	<0.1	<0.04	7	74	<2	<15	15	1.1	430.1	11.5	2300	1.18	143.96	0.24	55.2
6	Desert Pavement	10	1.083	Y	1.22	8.1	0.43	0.15	11.5	0.5	0.05	1	141	5	30	45	3.95	1544.45	39.8	7960	1.07	130.54	0.4	92
6	Desert Pavement	20	<0.067	*Y*	1.29	7.8	2.51	0.88	44.9	0.6	<0.04	1	0	-	-	163	7.33	2866.03	153.4	30680	1.2	146.4	0.62	142.6
6	Desert Pavement	30	<0.067	*Y*	1.46	7.8	-	-	29.1	0.4	<0.04	1	0	-	-	76	7.07	2764.37	68	13600	0.57	69.54	0.81	186.3
6	Desert Pavement	40	<0.067	N	1.29	8.2	3.95	1.38	1.9	<0.1	<0.04	2	0	-	-	172	5.14	2009.74	153.1	30620	0.86	104.92	12.91	2969.3
6	Desert Pavement	50	<0.067	N	1.48	8.7	3.39	1.19	0.8	<0.1	0.05	<1	1,467	<2	<15	47	4.8	1876.8	22.3	4460	0.31	37.82	19.29	4436.7
6	Desert Pavement	80	<0.067	N	1.33	8.2	-	-	0.5	0.1	0.26	1	0	-	-	174	4.74	1853.34	131.4	26280	0.98	119.56	37	8510
8	Playa	0	0.661	Y	0.99	7.8	2.26	0.79	13.6	0.2	<0.04	4	1,940	<2	43	121	0.76	297.16	119.9	23980	0.39	47.58	0.24	55.2
8	Playa	10	0.597	Y	0.89	7.8	2.3	0.8	12.4	0.2	<0.04	2	1,992	<2	41	193	1.78	695.98	189.7	37940	0.99	120.78	0.22	50.6
8	Playa	20	<0.067	N	0.96	8	2.43	0.85	9.6	0.2	<0.04	2	1,131	<2	39	84	2.56	1000.96	77.6	15520	2.33	284.26	1.37	315.1
8	Playa	30	<0.067	Y	1.12	7.9	2.78	0.97	11.8	0.2	<0.04	1	1,690	<2	34	124	1.7	664.7	117.3	23460	1.7	207.4	3.49	802.7
8	Playa	40	ND	N	1.04	7.9	2.78	0.97	6.5	0.2	<0.04	2	949	<2	35	94	1.91	746.81	87.1	17420	1.62	197.64	3.75	862.5
8	Playa	50	<0.067	Y	0.98	8	2.85	1	7.1	0.2	<0.04	1	1,393	<2	37	119	2.1	821.1	109.3	21860	1.93	235.46	5.69	1308.7
8	Playa	60	NT	*Y*	0.95	8	3.29	1.15	6.5	0.2	<0.04	1	1,766	<2	29	118	1.89	738.99	105.7	21140	1.34	163.48	9.28	2134.4
8	Playa	70	<0.067	*Y*	0.96	8.1	4.11	1.44	32.2	1.2	<0.04	1	2,350	<2	31	102	1.68	656.88	83	16600	1.09	132.98	16.51	3797.3
8	Playa	80	<0.067	*Y*	1.2	8.4	4.28	1.5	5.4	0.1	<0.04	1	2,370	<2	16	128	0.91	355.81	108.8	21760	0.22	26.84	18.04	4149.2
9	Playa	0	<0.067	Y	1.41	7.9	0.88	0.31	15.6	0.2	<0.04	2	449	<2	<15	26	0.52	203.32	25.1	5020	0.42	51.24	0.3	69
9	Playa	10	0.368	Y	1.46	7.8	2.17	0.76	22.1	0.2	<0.04	<1	1,018	<2	<15	65	0.25	97.75	64.9	12980	0.1	12.2	0.06	13.8
9	Playa	20	ND	N	1.07	7.9	2.28	0.8	11	0.2	<0.04	2	1,478	<2	21	137	1.51	590.41	134.6	26920	0.84	102.48	0.23	52.9
9	Playa	30	<0.067	N	1.41	7.8	2.24	0.78	12.7	0.2	<0.04	<1	1,226	57	<15	93	0.48	187.68	91.2	18240	0.67	81.74	0.22	50.6
9	Playa	40	<0.067	N	1.22	8	2.56	0.9	25.1	0.2	<0.04	1	1,338	103	<15	184	0.76	297.16	181.1	36220	0.58	70.76	1.49	342.7
9	Playa	50	NT	N	0.99	8	2.59	0.91	7.9	0.2	<0.04	<1	1,329	<2	34	150	1.97	770.27	144.3	28860	1.6	195.2	1.68	386.4
9	Playa	60	<0.067	N	1.48	7.8	2.26	0.79	22.8	0.2	<0.04	<1	1,036	<2	<15	60	0.53	207.23	58.5	11700	0.39	47.58	0.45	103.5
9	Playa	70	<0.067	N	1.23	7.7	2.32	0.81	14.9	0.2	<0.04	<1	1,508	<2	<15	97	0.77	301.07	94.5	18900	0.65	79.3	0.69	158.7
9	Playa	80	ND	N	1.48	7.6	2.25	0.79	7.2	0.1	<0.04	<1	1,145	16	<15	104	0.45	175.95	102.6	20520	0.37	45.14	0.42	96.6
11	Playa	0	<0.067	Y	1.17	8.1	0.87	0.3	6.8	0.2	<0.04	2	321	<2	30	37	1.26	492.66	34.8	6960	0.61	74.42	0.17	39.1
11	Playa	10	0.837	Y	1.13	7.9	2.33	0.82	10.3	0.2	<0.04	1	916	<2	19	63	1.39	543.49	61	12200	0.58	70.76	0.42	96.6
11	Playa	20	0.331	Y	1.24	7.9	2.5	0.88	34.7	0.5	<0.04	2	676	<2	21	51	1.42	555.22	47.3	9460	1.22	148.84	0.95	218.5
11	Playa	30	0.485	Y	1.29	8	2.98	1.04	5.6	0.1	<0.04	<1	1,266	<2	18	63	1.3	508.3	54.3	10860	1.51	184.22	5.83	1340.9
11	Playa	40	<0.067	N	1.41	7.8	2.83	0.99	3.3	<0.1	<0.04	<1	1,082	<2	<15	33	0.56	218.96	27.2	5440	0.81	98.82	4.01	922.3
11	Playa	50	0.293	N	1.2	7.9	2.61	0.91	4	0.1	<0.04	1	633	<2	<15	57	1.17	457.47	53.9	10780	0.73	89.06	0.86	197.8
11	Playa	60	<0.067	N	1.35	7.9	1.74	0.61	6.1	<0.1	<0.04	<1	473	<2	<15	28	0.78	304.98	26.3	5260	0.67	81.74	0.6	138
11	Playa	70	0.315	N	1.26	8.1	2.05	0.72	7.2	<0.1	<0.04	<1	543	<2	<15	35	0.92	359.72	32.1	6420	0.78	95.16	1.26	289.8
11	Playa	80	<0.067	N	1.57	7.9	2.15	0.75	8.1	<0.1	<0.04	<1	943	<2	<15	34	0.33	129.03	32.9	6580	0.23	28.06	0.85	195.5
10	Paleo-playa	0	ND	N	0.96	7.8	2.35	0.82	10.6	0.1	<0.04	3	2,210	<2	32	179	0.62	242.42	177.2	35440	0.23	28.06	0.87	200.1
10	Paleo-playa	10	ND	N	1.08	7.8	2.29	0.8	30.5	0.3	<0.04	<1	1,485	8	<15	263	1.34	523.94	261	52200	0.39	47.58	0.18	41.4
10	Paleo-playa	20	0.581	N	1.17	7.7	2.45	0.86	9.5	0.1	<0.04	<1	1,930	<2	<15	235	3.4	1329.4	231	46200	0.75	91.5	0.33	75.9
10	Paleo-playa	30	<0.067	N	1.11	8.5	6.07	2.12	14.7	0.2	<0.04	<1	3,330	146	45	254	8.13	3178.83	226	45200	1.02	124.44	18.59	4275.7
10	Paleo-playa	40	<0.067	N	1.25	8.6	9.4	3.29	1.8	0.1	0.07	<1	3,890	4	39	240	2.79	1090.89	200	40000	0.9	109.8	36.2	8326
10	Paleo-playa	50	<0.067	N	1.16	8.1	15.49	5.42	<0.5	0.1	0.46	<1	2,930	132	32	258	2.6	1016.6	185.5	37100	0.62	75.64	69.3	15939
10	Paleo-playa	60	<0.067	N	1.21	8.1	9.66	3.38	<0.5	<0.1	0.21	<1	2,110	<2	27	163	1.84	719.44	119.4	23880	0.56	68.32	41.4	9522
10	Paleo-playa	70	NT	N	1.07	8	8.82	3.09	0.7	<0.1	0.05	<1	2,770	<2	30	192	2.26	883.66	159.7	31940	0.56	68.32	29.1	6693
10	Paleo-playa	80	<0.067	N	1.14	8	8.75	3.06	0.8	0.1	0.17	1	2,400	134	<15	258	1.83	715.53	225	45000	0.45	54.9	30.7	7061

Table S4. Sediment microclimate variables for representative desert pavement and playa sites.

a) Comparison of gravimetric moisture content in pavement and playa sediment depth horizons.

Depth	**Desert pavement Site 1 (%)**	**Playa Site 8 (%)**
0 cm	0.12	2.3
10 cm	1.7	10.1
20 cm	1.2	8.8
30 cm	2.0	13.5
40 cm	3.5	15.0
50 cm	3.5	16.6
80 cm	2.2	8.7

b) Summary of depth horizon microclimate variables from *in situ* dataloggers deployed at desert pavement Site 1 at the time of sediment sampling for 14 months (note: playa Site 8 dataloggers were lost as a result of unusual heavy rainfall). A full time series for these data are shown in Fig. S6.

**Variable**	**10 cm**	**30 cm**	**80 cm**
Average Temp °C	19.9	20.3	20.9
Max Temp °C	34.4	26.1	25.3
Min Temp °C	3.2	14.7	20.0
Average %RH	22.6	20.4	22.6
Max %RH	97.7	31.2	23.6
Min %RH	9.8	16.8	20.9
hrs./yr. liquid water equivalent	304	Approx. 0	Approx. 0

Table S5. Environmental DNA recovery from sediments recovered using the rover-mounted drill (DNA recovery for manual samples is shown in Table S2).

Site	Sample depth (cm)	Site Type	DNA ng/g
1	10-20	Desert Pavement	0.315
	30-47	Desert Pavement	4.667
	47-58	Desert Pavement	0.312
	58-68	Desert Pavement	0.789
	68-77	Desert Pavement	<0.067
2	Surface	Desert Pavement	<0.067
	20-46	Desert Pavement	6.4
6	Surface	Desert Pavement	<0.067
	10	Desert Pavement	<0.067
	30	Desert Pavement	<0.067
	80	Desert Pavement	<0.067
8	Surface	Playa	<0.067
	17-30	Playa	<0.067
	30-60	Playa	<0.067
	60-80	Playa	<0.067
	>60-80	Playa	<0.067
9	Surface	Playa	<0.067
	0-30	Playa	1.4
	30-60	Playa	<0.067
10	Surface	Playa	<0.067
	0-24	Playa	<0.067
	0-30	Playa	<0.067
	10-30	Playa	<0.067
	24-71	Playa	0.315
	40-80	Playa	<0.067
11	Surface	Playa	<0.067
	0-10	Playa	<0.067
	0-30	Playa	0.597
	10-30	Playa	0.629
	0-55	Playa	0.848
	33-63	Playa	<0.067
	77-80	Playa	0.277

Table S6. Sequencing read depth and alpha diversity metrics for successfully sequenced sediment samples.

Site	Depth (cm)	Manual (M)/Drill (D)	Sequencing read depth	Species Richness (Chao 1)	Shannon's Index	Simpson Index	Pielou's Evenness
1	0	M	8149	164	3.009	0.016	0.611
1	10	M	16472	213	3.429	0.029	0.677
1	20	M	33364	166	2.723	0.009	0.540
1	10 to 20	D	11899	259	3.752	0.029	0.694
1	30 to 47	D	33176	303	3.674	0.024	0.652
1	47 to 58	D	9281	260	3.522	0.021	0.652
1	58 to 68	D	9293	247	3.556	0.022	0.659
2	0	M	16860	293	3.052	0.012	0.549
2	0	D	18253	122	2.022	0.007	0.440
2	10	M	4324	177	3.807	0.040	0.757
2	20 to 46	D	3312	221	3.986	0.036	0.756
2	20	M	11048	177	2.894	0.010	0.579
2	30	M	9702	107	2.687	0.014	0.600
6	0	M	12496	123	3.113	0.023	0.661
6	0	D	49435	116	2.088	0.006	0.445
6	0	D	44735	155	2.284	0.008	0.467
6	0	D	47874	141	2.374	0.009	0.494
6	10	M	12088	135	3.023	0.019	0.636
6	10	D	31461	176	3.031	0.019	0.611
6	30	D	51539	94	0.426	0.002	0.097
8	10	M	5542	197	3.604	0.024	0.703
8	30	M	26198	196	3.519	0.026	0.676
8	50	M	83654	86	0.357	0.002	0.085
8	60 to 80	D	44442	174	2.076	0.005	0.407
8	70	M	65082	85	0.586	0.002	0.134
9	0	M	39729	98	2.108	0.007	0.484
11	0	M	39774	210	2.863	0.014	0.559
11	0	D	22482	134	2.511	0.010	0.526
11	20	M	46333	207	2.667	0.009	0.521
11	0 to 30	D	32794	322	3.980	0.046	0.704
